# Delay in breast cancer surgery: evaluating patient, healthcare access, and social vulnerability predictors

**DOI:** 10.1007/s10552-026-02127-2

**Published:** 2026-02-10

**Authors:** Melanie Boyd, Mandana Rezaeiahari, Mario Schootman, Yong-Moon Park, Kelsey M. Owsley

**Affiliations:** 1 University of Arkansas for Medical Sciences College of Public Health, Little Rock, AR USA; 2 University of Arkansas for Medical Sciences College of Medicine, Little Rock, AR USA; 3 University of Arkansas for Medical Sciences Winthrop P. Rockefeller Cancer Institute, Little Rock, AR USA; 4 University of Arkansas for Medical Sciences College of Nursing, Little Rock, AR USA; 5 Department of Health Policy and Management, Fay W. Boozman College of Public Health, 4301 W. Markham St., #820, Little Rock, AR 72205-7199 USA; 6 Department of Epidemiology, Fay W. Boozman College of Public Health, 4301 W. Markham St., #820, Little Rock, AR 72205-7199 USA

**Keywords:** Breast cancer, Time to surgery, Healthcare access, Timely oncology treatment, Disparities, Social vulnerabilities

## Abstract

**Purpose:**

Clinical guidelines recommend initiating breast cancer treatment within 60 days of diagnosis. We examined the extent to which patient characteristics, healthcare access, and social vulnerability are associated with delay in surgical treatment among breast cancer patients in Arkansas.

**Methods:**

We used 2013–2019 data from the linked Arkansas Central Cancer Registry and the Arkansas All-Payer Claims Database (APCD) to identify female patients with breast cancer who received first-line surgical treatment (*n* = 6,279). Time to surgery (TTS) was defined as the interval between diagnosis and first surgical treatment and dichotomized at 60 days. The American Hospital Association Survey and AHRQ’s Social Determinants of Health Database captured hospital- and community-level characteristics of healthcare access. We applied multivariable logistic regression models to assess associations between TTS and predictor variables.

**Results:**

Overall, 12% of patients received surgery after 60 days of diagnosis. Non-Hispanic Black patients had 82% higher odds of surgical delay (adjusted odds ratio [aOR] 1.82; 95% confidence interval [CI] 1.46–2.29) compared to non-Hispanic White patients. Residing in counties with high social vulnerability index scores for housing/transportation (aOR 1.38; 95% CI 1.06–1.81) was associated with greater odds of delay, whereas higher county-level rates of routine doctor visits (aOR 0.89; 95% CI 0.82–0.97) were associated with lower odds of delays.

**Conclusion:**

Disparities in delay in breast cancer surgery persist across race, healthcare access, and community-level vulnerability. These findings underscore the need for strengthened support services and improved continuity of care among vulnerable populations.

**Supplementary Information:**

The online version contains supplementary material available at 10.1007/s10552-026-02127-2.

## Introduction

Around 13% of females in the USA develop breast cancer at some point in their lifetime [1]. Despite advances in treatment, 8.3% of patients diagnosed with breast cancer die within 5 years [2]. Timely treatment is critical for improving survival among breast cancer patients, as patients who experience surgical delay of more than 60 days following diagnosis face a 26% higher mortality risk [3]. Recognizing the importance of timely care, the American Society of Breast Surgeons (ASBrS) endorses timeliness as a national quality measure reporting the percentage of newly diagnosed breast cancer patients who initiate treatment within 60 days of their diagnostic biopsy [4].

In Arkansas, female breast cancer was the second most diagnosed cancer from 2015 to 2019 [5], and disparities in cancer outcomes persist across geographic, demographic, and socioeconomic groups [5, 6]. Access to cancer care remains a significant challenge in the state, which is consistently ranked at the bottom for healthcare system performance [7]. Access to care is critical for cancer patients as routine care provides opportunities for patients to discuss symptoms, receive cancer screening recommendations, and obtain referrals for timely treatment [8]. Additionally, unmet social needs and community-level vulnerabilities, such as poverty, unemployment, lack of health insurance, housing instability, and unreliable transportation, impose significant barriers to accessing cancer screening and treatment [9, 10]. Despite these challenges, few studies have examined timeliness of treatment in the context of local healthcare availability and community-level vulnerability.

In this study, we examine the association between delayed breast cancer surgery and patient, healthcare access, and community-level social vulnerability characteristics among Arkansas breast cancer patients. This study builds on prior literature by incorporating measures of healthcare infrastructure, such as availability of hospital-based oncology and surgical services, and structural barriers that may contribute to treatment delays [3, 11–17]. The results of this study provide insight to inform efforts to improve access to timely surgical care.

## Methods

### Data

We identified breast cancer patients from the Arkansas Central Cancer Registry (ACCR), a gold-standard registry designated by the North American Association of Central Cancer Registries (NAACCR) and linked these patients to medical claims from the Arkansas All Payers Claims Database (APCD) [18, 19]. The APCD is a health insurance administrative database that houses billing claims and enrollment information for members of state and federal health plans, and certain private market health plans overseen by the Arkansas Center for Health Improvement (ACHI) [20]. The ACCR is known to have incomplete treatment information; therefore, the linked ACCR-APCD offers a more comprehensive source of data for analysis [21]. Studies have found that the Arkansas APCD provides high sampling coverage among cancer patients and is broadly representative of the state’s population [22, 23]. We used data from the American Hospital Association’s (AHA) annual hospital survey to measure hospital-based oncology and surgical service availability aggregated to the county level. The Agency for Healthcare Research and Quality (AHRQ) Social Determinants of Health Database provided county-level information on the proportion of the population with routine physician visits and social vulnerability indicators [24].

### Study population

Arkansas adults aged 18 years and older with an initial diagnosis of breast cancer reported in ACCR from 2013 to 2019 comprised the study population (*n* = 14,770). Initial diagnoses were defined as having a first lifetime neoplasm with breast as the primary site. Patients with incomplete cancer staging information or missing diagnosis dates were excluded (*n* = 2,262). We linked the remaining breast cancer patients (*n* = 12,508) to APCD administrative claims using a unique identifier created and de-identified by ACHI. We further excluded patients without claims data, without documented breast cancer treatment in the claims, or with treatment at the time of diagnosis or more than 180 days following diagnosis (*n* = 5,674). Treatment at the time of diagnosis are possible excisional biopsies or cancer discovered secondarily [17]. Intervals longer than 180 days are shown to be associated with comorbidities or treatment for secondary tumors [25, 26]. The sample included only women (excluding male patients, *n* = 41) whose first-line treatment following diagnosis was surgery (excluding patients with other first-line treatments including chemotherapy, radiation, and neoadjuvant therapy, *n* = 514). The final analytic sample comprised 6,279 breast cancer patients.

### Variables

Our primary outcome was time to surgical treatment (TTS), defined as the number of days between the breast cancer diagnosis date and the date of subsequent breast cancer surgery. The date of breast cancer diagnosis was obtained from the ACCR and reflects the date a medical professional clinically or microscopically confirmed cancer [27, 28]. Surgical treatments for breast cancer were identified using inpatient and outpatient medical claims and procedure codes from the Healthcare Common Procedure Coding System (HCPCS), Current Procedural Terminology (CPT), and International Classification of Diseases, Ninth and Tenth Revision (ICD-9/10) for breast cancer surgery (see Table S1 for details) [29]. Surgery dates were identified as the procedures occurring immediately after, but not on, the date of diagnosis. TTS was dichotomized at a 60-day threshold in accordance with the ASBrS timeliness of care measure. Delayed treatment was defined as patients who received surgery after 60 days.

The analysis included potential predictor variables for patient characteristics and county-level measures of healthcare access and social vulnerability. We selected patient-level variables associated with treatment delay, while county-level measures were included to account for structural factors that may influence timely treatment [13, 15, 17]. Patient characteristics included age (18–49, 50–64, 65–74, and 75 +), race/ethnicity (non-Hispanic White, non-Hispanic Black, Hispanic and other race), insurance (private, Medicaid, Medicare, and dual Medicare/Medicaid), rurality, cancer stage (localized/in situ vs. regional/distant), triple-negative diagnosis, receipt of mastectomy, and diagnosis year. Age groups were determined based on considerations, such as clinical screening recommendations and Medicare eligibility. While more granular reporting of race and ethnicity is advantageous, only 2% of patients comprised the ‘other race’ category which would results in small cell sizes if further disaggregation were reported. A Rural Urban Commuting Area (RUCA) code greater than 3 defined rurality. County-level healthcare access variables included the percentage of adults with a routine doctor visit and dichotomous indicators for whether the county had a hospital offering oncology and surgical services (outpatient or inpatient), and the presence of a Commission on Cancer (CoC)-accredited cancer program. County-level social vulnerability was measured using four composite index variables from the Centers for Disease Control and Prevention and Agency for Toxic Substances and Disease Registry’s Social Vulnerability Index (SVI) [30]. The SVI is organized into four domains: socioeconomic status, household characteristics, racial and ethnic minority status, and housing type and transportation. Higher SVI scores reflect greater county-level vulnerability. We dichotomized the SVI variables to indicate whether a county fell within the top quartile for each domain based on the sample distribution of patient’s county of residence.

### Statistical analysis

We used multivariable logistic regression to estimate predictors of breast cancer surgical delay, measured as TTS greater than 60 days. We also conducted subgroup analyses stratified by breast cancer stage at diagnosis (in situ/localized vs. regional/distant). Patients with greater clinical complexity often require specialized services, which may increase the risk of surgical delay [31]. In contrast, those with earlier-stage disease are more likely to undergo breast-conserving treatment, which typically does not require coordination between breast and reconstructive surgical teams and can be scheduled more quickly [32]. We also estimated a fully interacted model to assess whether differences varied significantly by cancer stage. In sensitivity analyses, we examined a 45-day threshold, another commonly used benchmark to assess the robustness of findings [31]. All models included calendar year fixed effects to adjust for and examine temporal trends and used county-clustered standard errors. Data preparation and analysis were performed using SAS 9.4 (SAS Institute Inc., Cary, NC). The *p* values are two-sided, with the level of significance at 0.05. The study was determined to be exempt by the first author’s Institutional Review Board.

## Results

Characteristics of breast cancer patients for the total sample and by TTS (≤ 60 days vs. > 60 days) are presented in Table 1. About half of the sample were aged 65 years or older (57.2%), non-Hispanic White (85.1%), insured privately (47.9%) or through Medicare (42.4%), and lived in an urban county (59.7%). Around 21% of the breast cancer cases were classified as advanced stage, 33% had a mastectomy rather than breast-conserving surgery and 8.2% were diagnosed as triple negative. More than half of patients lived in counties with an oncology hospital (53.1%) or a hospital offering surgical inpatient or outpatient services (84.0%), while only 8.6% lived in a county with a CoC facility. Of the 6,279 in the total sample, 12% underwent breast surgery more than 60 days after diagnosis.

**Table 1:** Breast patient characteristics by time to surgery in Arkansas, 2013–2019

Total sample, *n* (%)	Total sample	Time to surgery in days		*p* value
		≤ 60	> 60	
	6,279 (100.0%)	5,501 (87.6%)	778 (12.4%)	
Patient-level characteristics				
Age				< 0.0001
18–49	745 (11.9%)	580 (10.5%)	165 (21.2%)	
50–64	1,940 (30.9%)	1,679 (30.5%)	261 (35.5%)	
65–74	2,300 (36.6%)	2,049 (37.3%)	251 (32.3%)	
75 and older	1,294 (20.6%)	1,193 (21.7%)	101 (13.0%)	
Race/ethnicity				< 0.0001
Non-Hispanic White	5,345 (85.1%)	4,723 (85.9%)	622 (80.0%)	
Non-Hispanic Black	810 (12.9%)	669 (12.1%)	141 (18.1%)	
Hispanic/other race	124 (2.0%)	109 (2.0%)	15 (1.9%)	
Insurance				< 0.0001
Private	3,005 (47.9%)	2,581 (46.9%)	424 (54.5%)	
Medicaid	306 (4.9%)	244 (4.4%)	62 (8.0%)	
Medicare	2,664 (42.4%)	2,413 (43.9%)	251 (32.2%)	
Medicare/Medicaid	304 (4.8%)	263 (4.8%)	41 (5.3%)	
Cancer stage				< 0.0001
In situ/localized	4,989 (79.5%)	4,417 (80.3%)	572 (73.5%)	
Regional/distant	1,290 (20.5%)	1,084 (19.7%)	206 (26.5%)	
Rural				0.13
Yes	2,530 (40.3%)	2,236 (40.7%)	294 (37.8%)	
No	3,749 (59.7%)	3,265 (59.3%)	484 (62.2%)	
Triple negative				0.83
Yes	513 (8.2%)	451 (8.2%)	62 (8.0%)	
No	5,766 (91.8%)	5050 (91.8%)	716 (92.0%)	
Mastectomy				< 0.0001
Yes	2,076 (33.1%)	1,625 (29.4%)	451 (58.0%)	
No	4,203 (66.9%)	3,876 (70.5%)	327 (42.0%)	
County-level characteristics				
% Adults with routine doctor visit, mean	77.0 (0.02)	77.0 (0.02)	76.9 (0.06)	0.37
Oncology hospital				0.88
Yes	3,333 (53.1%)	2,918 (53.0%)	415 (53.3%)	
No	2,946 (46.9%)	2,583 (47.0%)	363 (46.7%)	
Surgical hospital				0.09
Yes	5,276 (84.0%)	4,606 (83.7%)	670 (86.1%)	
No	1,003 (16.0%)	895 (16.3%)	108 (13.9%)	
Commission on cancer hospital				0.21
Yes	540 (8.6%)	464 (8.4%)	76 (9.8%)	
No	5,739 (91.4%)	5,037 (91.6%)	702 (90.2%)	
Socioeconomic SVI ranking				0.93
< Q3	4,721 (75.2%)	4,135 (75.2%)	586 (75.3%)	
≥ Q3	1,558 (24.8%)	1,366 (24.8%)	192 (24.7%)	
Household SVI ranking				0.39
< Q3	4,632 (73.8%)	4,068 (74.0%)	564 (72.5%)	
≥ Q3	1,647 (26.2%)	1,433 (26.0%)	214 (27.5%)	
Minority status SVI ranking				0.06
< Q3	4,546 (72.4%)	4,005 (72.8%)	541 (69.5%)	
≥ Q3	1,733 (27.6%)	1,496 (27.2%)	237 (30.5%)	
Housing/transportation SVI ranking				< 0.0001
< Q3	4,643 (74.0%)	4,114 (74.8%)	529 (68.0%)	
≥ Q3	1,636 (26.0%)	1,387 (25.2%)	249 (32.0%)	

Table values are presented as number (percentage) unless otherwise noted as mean (standard deviation). *p* values were calculated using Pearson chi-square and Student’s *t* tests to assess associations by time to surgery. Q = Quartile

In unadjusted analyses, patients with surgical delay were younger (age 18–49 years: 21.2% vs. 10.5%), Black (18.1% vs. 12.1%), and insured through a private payer (54.5% vs. 46.9%) or Medicaid (8.0% vs. 4.4%) (all *p* < 0.001). They also had higher rates of later-stage cancer (26.5% vs. 19.7%) and mastectomy (58.0% vs. 29.4%) (all *p* < 0.001). Additionally, a greater proportion of patients with surgical delay lived in areas ranked in the top SVI quartile for housing and transportation (32.0% vs. 25.2%; *p* < 0.001) and approaching statistical significance for minority status (30.5% vs. 27.2%; *p* = 0.06).

**Fig. 1:**
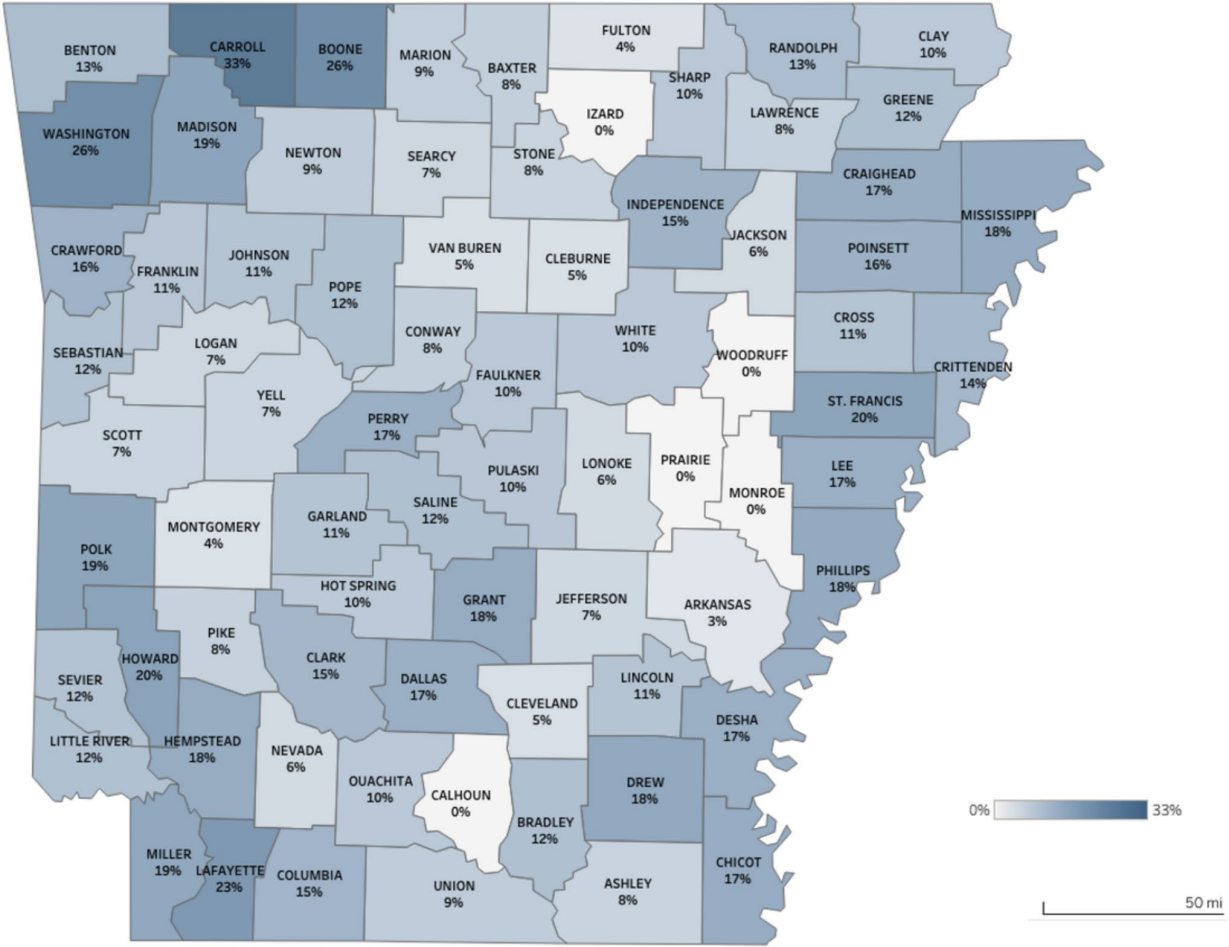
Percentage of breast cancer patients with time to surgery greater than 60 days by county

Figure 1 illustrates that surgical delay varied geographically. Carroll County had nearly one-third of patients experiencing surgical delay (33%), followed by Boone (26%) and Washington (26%) counties, all located in the northwest region of the state. Surgical delays in the northwest region may be due to several factors. The northwest region generally has a younger population and a higher proportion of privately insured residents. Patients in this area may also face longer traveling distances if seeking care in the central part of the state when referred to high volume cancer care centers. The central region, the most metropolitan area of the state, had some of the lowest proportions of surgical delay. In contrast, the Delta region, a highly rural, persistently poor area in eastern Arkansas with a large proportion of racial and ethnic minorities, exhibited wide variation, ranging from 0% in counties such as Monroe and Prairie to 20% in St. Francis County. The number of breast cancer patients in each county can be found in the Supplementary Information (Fig. S1).

**Fig. 2:**
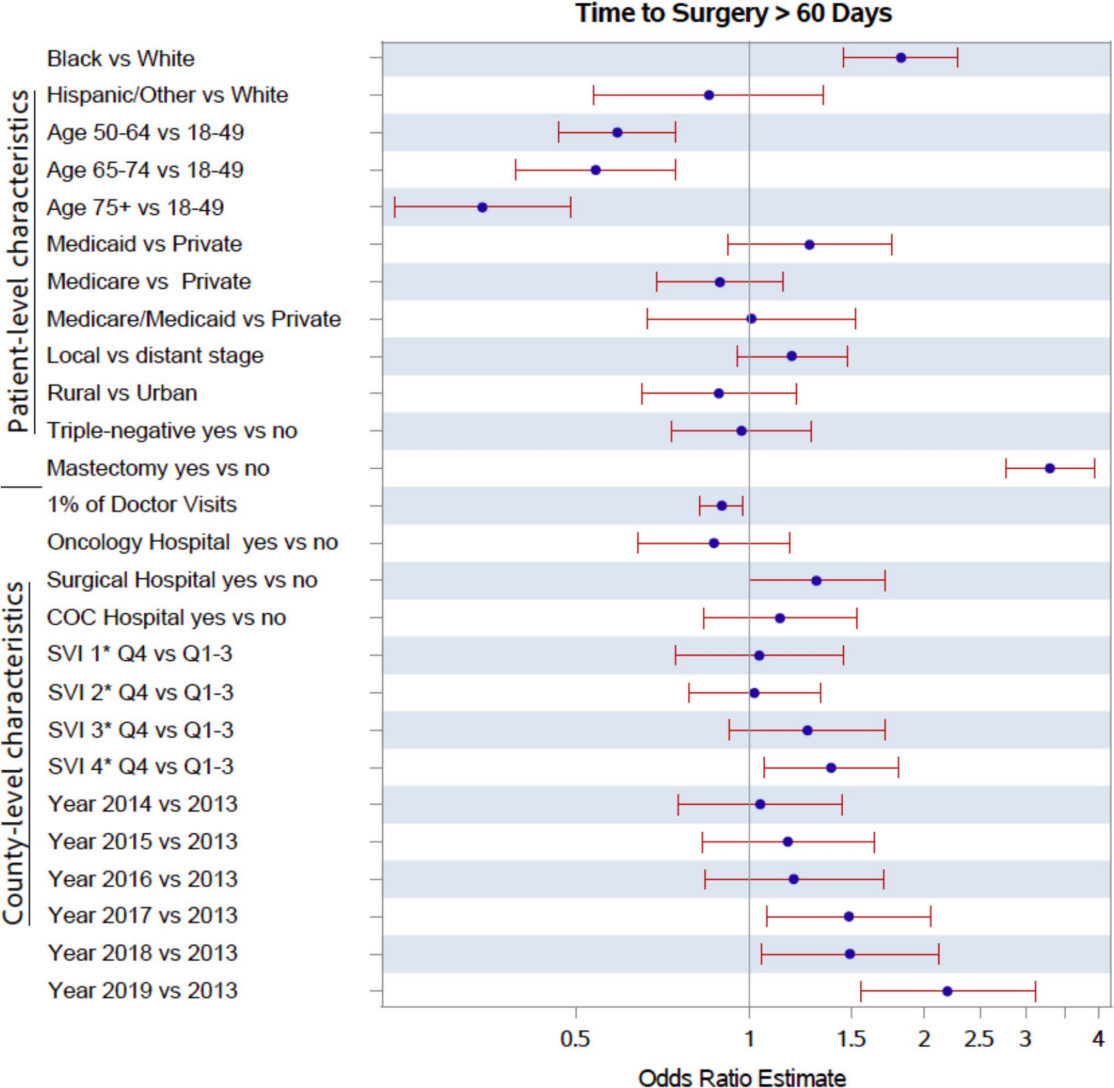
Characteristics associated with surgical delay. Analyses used logistic regression with standard errors clustered at the county levels. Estimates are represented on a logarithmic scale. SVI 1, socioeconomic status; SVI 2, household characteristics; SVI 3, racial and ethnic minority status; SVI 4, housing type and transportation. SVI = Social Vulnerability Index

**Fig. 3:**
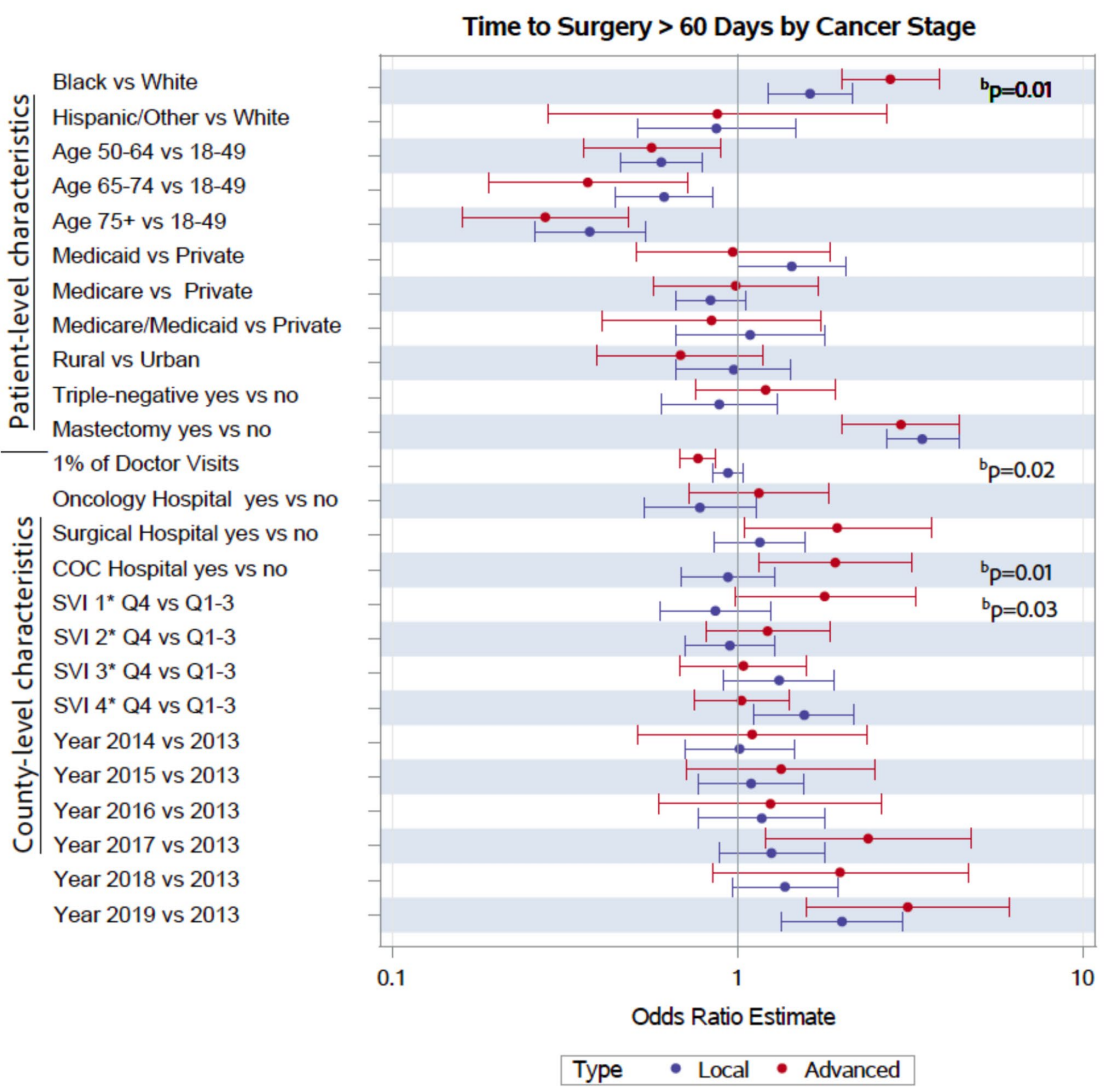
Characteristics associated with surgical delay by cancer stage. Analyses used logistic regression with standard errors clustered at the county levels. Estimates are represented on a logarithmic scale. SVI 1, socioeconomic status; SVI 2, household characteristics; SVI 3, racial and ethnic minority status; SVI 4, housing type and transportation. SVI = Social Vulnerability Index. ^b^Indicates significantly different cancer stage interaction term using Wald *p* values

Figures 2 and 3 present adjusted odds ratios (aOR) for predictors of surgical delay. Breast cancer patients aged 75 years and older (aOR 0.35; 95% confidence interval [CI] 0.24–0.49), those aged 65–74 (aOR 0.54; 95% CI 0.39–0.74), and those aged 50–64 (aOR 0.59; 95% CI 0.47–0.75) had 65%, 46%, and 41% lower odds, respectively, of experiencing surgical delay compared to patients aged 18–45 years. Black patients had 82% higher odds of surgical delay (aOR 1.82; 95% CI 1.46–2.29) compared to their White counterparts. Patients undergoing mastectomy had significantly higher odds of surgical delay compared to those receiving breast-conserving surgery (aOR 3.31; 95% CI 2.77–3.95). No statistically significant differences were observed by insurance status, cancer stage, triple-negative diagnosis, or rurality in adjusted models. A 1% increase in the percentage of adults receiving routine doctor visits were associated with lower odds of surgical delay (aOR 0.89; 95% CI 0.82–0.97). Residing in a county with a hospital offering oncology services had reduced odds of delay (aOR 0.87; 95% CI 0.64–1.17), although this association was not statistically significant. In contrast, patients living in counties with hospitals providing inpatient or outpatient surgical services had higher odds of surgical delay approaching statistical significance (aOR 1.31; 95% CI 1.00–1.71).

Patients living in counties in the top SVI quartile (i.e., most vulnerable counties) for housing/transportation vulnerability (aOR 1.38; 95% CI 1.06–1.81) had higher odds of experiencing surgical delay. No significant differences were observed for counties in the top quartile for socioeconomic, minority status, or household vulnerability. Additionally, patients diagnosed in later years had increased odds of surgical delay. Compared with 2013, diagnoses in 2017 (aOR 1.48; 95% CI 1.07–2.05), 2018 (aOR 1.49; 95% CI 1.05–2.12), and 2019 (aOR 2.20; 95% CI 1.55–3.11) were associated with higher odds of surgical delay (see Table S2).

Figure 3 presents results from sub-analyses stratified by cancer stage. Among patients diagnosed with localized/in situ breast cancer, 11% received surgery after 60 days of diagnosis, while 16% of patients diagnosed with regional/distant disease had delayed surgery. We observed similar patterns across most predictors; however, a few notable differences existed. Among Black patients, those with advanced cancer had higher odds of surgical delay than those with localized cancers (*p* = 0.01). Medicaid insured patients had significantly higher odds of surgical delay among those with localized disease, but not for those with advanced disease. However, the association was not significantly different by cancer stage using interacted models (*p* = 0.28). Patients residing in counties with a higher proportion of the population receiving routine doctor visits had significantly lower odds of delayed treatment for those with advanced cancer versus localized cancers (*p* = 0.02). Patients residing in counties with a COC hospital or high socioeconomic vulnerability had significantly higher odds of surgical delay among those diagnosed with advanced cancer compared to those with localized disease (all *p* < 0.05). In sensitivity analyses using a 45-day threshold for TTS, results were highly robust to the 60-day threshold outcome (see Table S3).

## Discussion

This study examined patient, healthcare access, and community vulnerability characteristics among Arkansas breast cancer patients from 2013 to 2019 to identify factors associated with surgical delay. Overall, we found 12% of patients statewide received surgery beyond 60 days with substantial geographic variation in delay across Arkansas, as well as a concerning trend of increasing treatment delays over time. Younger patients, Black women, and those residing in counties with high social vulnerabilities related to housing/transportation access had higher odds of experiencing surgical delays exceeding 60 days. Measures of healthcare access were also significantly associated with TTS. Patients residing in counties with higher rates of routine doctor visits had lower odds of surgical delay. In contrast, patients living in counties with hospitals offering surgical services were associated with higher odds of surgical delay.

Our findings confirm that Black patients experience significant treatment delays compared with White patients [11, 13], and that older patients have lower odds of treatment delays than younger patients [13]. In contrast to prior studies, we did not observe a significant delay in treatment among patients insured by Medicaid compared to those with private insurance [16]. However, in stratified analyses, Medicaid coverage was associated with increased odds of surgical delay among patients with localized stage cancer. This finding may reflect persistent access barriers for Medicaid patients. Medicaid’s historically lower reimbursement rates, compared to private or Medicare insurance, have limited access to primary and specialty care by reducing appointment availability or leading some providers to not accept Medicaid patients altogether [33]. Additionally, prior studies found significant surgical delays for patients living in urban areas [16, 34]. However, we did not find a significant delay in treatment by rurality. This discrepancy may reflect the inclusion of other predictors, such as limited healthcare availability and social vulnerability, which place rural residents at higher risk of delay.

We also found that patients living in counties with a higher proportion of adults reporting routine doctor visits were associated with a lower likelihood of surgical delay. Although this is a population-level measure, it may serve as a proxy for access to and engagement with regular healthcare in the area. Prior research has shown that regular visits with a primary care physician are associated with improved cancer outcomes, including shorter time to treatment [35, 36]. Counties with higher levels of regular healthcare engagement may have stronger care infrastructure, better care coordination, and more efficient navigation to specialty services [37], all of which support more timely cancer treatment.

Patients residing in counties with CoC-accredited hospitals experienced delayed treatment for advanced stage cancers, but this association was not observed overall. This finding aligns with prior studies reporting that patients living in low-income areas who receive care at CoC-accredited hospitals often experience treatment delays [38]. This may be due to patients having trouble navigating the healthcare system or not enough providers to meet the demand for treatment. Additionally, residing in counties with hospital-based surgical services was also associated with delayed breast cancer surgery. These results suggest that patients seeking care at high-quality facilities may face tradeoffs in treatment timeliness.

We also found that county-level social vulnerability was associated with increased surgical delay. Patients residing in areas with higher SVI scores for housing/transportation and socioeconomic status among early-staged and late-staged cancer patients, respectively, were more likely to receive care later than those in areas with lower SVI scores. These findings are consistent with recent studies reporting associations between SVI and treatment delays for lung cancer [39, 40]. Implementing social needs screening and interventions may improve cancer care coordination and address barriers related to social circumstances. Evidence suggests these programs are effective at linking patients to needed resources, although findings are mixed on improving health outcomes [41, 42].

Finally, we observed a concerning trend of increasing treatment delays in recent years. We suspect this trend is driven by a combination of factors. The growing incidence of breast cancer may be straining hospital and provider capacity to deliver timely surgical care [5]. The rapid consolidation and centralization of oncology practices may also contribute to treatment delays by increasing patient travel times [43]. While centralization can improve quality through provider specialization, it may also exacerbate disparities by assuming patients can access care in urban centers [44]. Lastly, among the Medicare-eligible population, the growing enrollment in Medicare Advantage may also contribute to treatment delays due to prior authorization requirements and narrow provider networks. Recent evidence has found that Medicare Advantage enrollment is associated with reduced access to cancer care [45, 46]. Although we did not examine Medicare Advantage enrollment separately in this study, it may be a potential driver of treatment delay.

### Limitations

Several limitations should be considered when interpreting these findings. First, the results are purely associational, and we cannot establish causal relationships between TTS and the predictor variables. Second, distance to treatment was not included in the analysis because treatment location information was unavailable for a considerable number of patients. Third, the findings are specific to patients living in Arkansas and may not be generalizable to populations outside the state. However, Arkansas has a large rural and racially diverse population, making it a critical state for efforts to reduce disparities in cancer outcomes. Fourth, social determinants of health and healthcare access are measured at the county level, which may mask important within-county geographic variation. Further assessment with nuanced individual level data is needed. Fifth, data submission to the APCD is not mandated for small private (< 2,000 enrollees) and self-insured plans not receiving state funding. As a result, coverage is limited in some areas of the state, such as the Northwest region [23]. Sixth, because uninsured patients are not captured in billing claims data, we were unable to examine the association between lack of health insurance and time to breast cancer surgery. Patients who receive additional services such as reconstructive surgery and genetic testing may also contribute to extended time between diagnosis and surgery. Although it was outside the scope of the focus of this study, further investigations could contribute insights to the delay of treatment. Finally, statistical tests of interactions require large sample sizes, and limited power may prevent the detection of meaningful differences.

## Conclusion

Although most breast cancer patients in Arkansas received surgical treatment within the recommended 60-day window, disparities persist by race and community-level vulnerability. Routine doctor visits emerged as a factor associated with fewer treatment delays, which underscores the importance of strengthening access to primary and specialty care to support timely cancer treatment. Improving continuity of care and addressing barriers related to social vulnerabilities and healthcare access may help reduce disparities and expedite surgical treatment for breast cancer patients.

## Supplementary Information

Below is the link to the electronic supplementary material.

Supplementary file1 (DOCX 287 KB)

## Data Availability

Data for this study were obtained from the Arkansas Center for Health Improvement (ACHI) under a data use agreement. The data are available from ACHI for researchers who meet the criteria for access and enter into a data use agreement. The authors are not permitted to distribute the data.

## References

[1] National Breast Cancer Foundation Team, Shockney LD. Breast Cancer Facts & Stats. https://www.nationalbreastcancer.org/breast-cancer-facts/. Accessed 23 June 2025

[2] National Cancer Institute, Surveillance, Epidemiology, and End Results Program. Cancer Stat Facts: Female Breast Cancer. https://seer.cancer.gov/statfacts/html/breast.html. Accessed 27 Jan 2025

[3] Bleicher RJ, Ruth K, Sigurdson ER et al (2016) Time to surgery and breast cancer survival in the United States. JAMA Oncol 2(3):330–339 26659430 10.1001/jamaoncol.2015.4508 PMC4788555

[4] The American Society of Breast Surgeons (ASBrS) (2022) Timeliness of care for breast cancer quality measure. https://www.breastsurgeons.org/docs/statements/asbrs-qm-timeliness-of-care-for-breast-cancer.pdf

[5] Arkansas Department of Health (2024). Arkansas cancer facts & figures. Accessed 18 June 2025. https://healthy.arkansas.gov/wp-content/uploads/Arkansas-Cancer-Facts-Figures-2024-Booklet-compressed.pdf

[6] Arkansas Department of Health, Office of Minority Health & Health Disparities and Epidemiology Branch (2018) Disparities in malignant neoplasms (CANCER) mortality among Blacks in Arkansas. https://healthy.arkansas.gov/wp-content/uploads/2018_Cancer_Mortality_Disparity_Fact_Sheet.pdf

[7] The Commonwealth Fund Arkansas: a collection of resources on health system performance in Arkansas. https://www.commonwealthfund.org/datacenter/arkansas. Accessed 27 Aug 2025

[8] Baum LVM, Kline K, Smith CB (2020) The “new normal” of cancer treatment delays is nothing new. JAMA Netw Open 3(12):e2030507. 10.1001/jamanetworkopen.2020.30507 33315109 10.1001/jamanetworkopen.2020.30507

[9] Goel N, Lubarsky M, Hernandez AE et al (2024) Unmet social needs and breast cancer screening utilization and stage at presentation. JAMA Netw Open 7(2):e2355301. 10.1001/jamanetworkopen.2023.55301 38353954 10.1001/jamanetworkopen.2023.55301 PMC10867685

[10] D’Alessandro D, Appolloni L (2020) Housing and health: an overview. Ann Ig 32(5 Supple 1):17–26. 10.7416/ai.2020.3391 33146364 10.7416/ai.2020.3391

[11] Ashing-Giwa KT, Gonzalez P, Lim J-W et al (2010) Diagnostic and therapeutic delays among a multiethnic sample of breast and cervical cancer survivors. Cancer 116(13):3195–3204. 10.1002/cncr.25060 20564623 10.1002/cncr.25060

[12] Chen JC, Stover DG, Ballinger TJ et al (2024) Racial disparities in breast cancer: from detection to treatment. Curr Oncol Rep 26(1):10–20. 10.1007/s11912-023-01472-8 38100011 10.1007/s11912-023-01472-8

[13] Gorin SS, Heck JE, Cheng B, Smith SJ (2006) Delays in breast cancer diagnosis and treatment by racial/ethnic group. Arch Intern Med 166(20):2244–2252. 10.1001/archinte.166.20.2244 17101943 10.1001/archinte.166.20.2244

[14] Yao KA, Snyder RA (2024) Observations from breast patients reveal barriers to achieving timely care. ACS Am Coll Surg 109(5). https://www.facs.org/for-medical-professionals/news-publications/news-and-articles/bulletin/2024/may-2024-volume-109-issue-5/observations-from-breast-patients-reveal-barriers-to-achieving-timely-care/

[15] Sukniam K, Kasbi AA, Ashary MA et al (2022) Disparities in time to treatment for breast cancer. Anticancer Res 42(12):5813–5818. 10.21873/anticanres.16088 36456136 10.21873/anticanres.16088

[16] Verdone CG, Bayron JA, Chang C et al (2022) A tool to predict disparities in the timeliness of surgical treatment for breast cancer patients in the USA. Breast Cancer Res Treat 191(3):513–522. 10.1007/s10549-021-06460-9 35013916 10.1007/s10549-021-06460-9 PMC8747888

[17] Wiener AA, Hanlon BM, Schumacher JR, Vande Walle KA, Wilke LG, Neuman HB (2023) Reexamining time from breast cancer diagnosis to primary breast surgery. JAMA Surg 158(5):485–492. 10.1001/jamasurg.2022.8388 36857045 10.1001/jamasurg.2022.8388 PMC9979003

[18] Arkansas Department of Health (2025) Data from Arkansas Central Cancer Registry (ACCR). https://healthy.arkansas.gov/programs-services/data-statistics-registries/arkansas-cancer-registry/. Accessed 25 July 2025

[19] Arkansas Center for Health Improvement (ACHI) (2021) Arkansas APCD biennial report 2021. https://www.arkansasapcd.net/ReportsAndMaps/. Accessed 23 June 2025

[20] Hart K *Arkansas APCD* (2025) ACHI infographics for the Arkansas health transparency initiative. https://achiapcd.atlassian.net/wiki/spaces/HTIR/pages/2621505562/Infographics+Arkansas+APCD. Accessed 22 July 2025

[21] Li C, Malapati SJ, Guire JT, Hutchins LF (2023) Consistency between state’s cancer registry and all-payer claims database in documented radiation therapy among patients who received breast conservative surgery. JCO Clin Cancer Inform 7:e2200099 36724402 10.1200/CCI.22.00099 PMC10166563

[22] Li C, Peng C, DelNero P, Saini M, Schootman M (2024) Sampling coverage of the Arkansas all-payer claims database by County’s persistent poverty designation. Health Serv Res 59(4):e14342. 10.1111/1475-6773.14342 38880660 10.1111/1475-6773.14342 PMC11249802

[23] Li C, Peng C, DelNero P et al (2025) Investigating the coverage of the Arkansas All-Payer Claims Database for examining health disparities related to persistent poverty areas in colorectal cancer patients. Cancer Causes Control 36(1):27–44 39306812 10.1007/s10552-024-01918-9 PMC13064871

[24] Agency for Healthcare Research and Quality (AHRQ) (2013–2019). Data from Social determinants of health database. Rockville, MD

[25] Polverini AC, Nelson RA, Marcinkowski E et al (2016) Time to treatment: measuring quality breast cancer care. Ann Surg Oncol 23(10):3392–3402. 10.1245/s10434-016-5486-7 27503492 10.1245/s10434-016-5486-7

[26] McLaughlin JM, Anderson RT, Ferketich AK, Seiber EE, Balkrishnan R, Paskett ED (2012) Effect on survival of longer intervals between confirmed diagnosis and treatment initiation among low-income women with breast cancer. J Clin Oncol 30(36):4493–4500 23169521 10.1200/JCO.2012.39.7695 PMC3518728

[27] National Cancer Institute, Surveillance, Epidemiology, and End Results Program (2023) SEER research plus data description

[28] National Cancer Institute, Surveillance, Epidemiology, and End Results Program (2024) SEER Research Plus Data Description. https://seer.cancer.gov/data-software/documentation/seerstat/nov2023/TextData.FileDescription-nov2023.pdf. Accessed 28 July 2025

[29] Shih Y-CT, Xu Y, Bradley C, Giordano SH, Yao J, Yabroff KR (2022) Costs around the first year of diagnosis for 4 common cancers among the privately insured. JNCI J Natl Cancer Inst 114(10):1392–1399. 10.1093/jnci/djac141 36099068 10.1093/jnci/djac141 PMC9552304

[30] Centers for Disease Control and Prevention (CDC) (2013–2019) Data from CDC SVI documentation

[31] Dong J, Esham KS, Boehm L et al (2020) Timeliness of treatment initiation in newly diagnosed patients with breast cancer. Clin Breast Cancer 20(1):e27–e35. 10.1016/j.clbc.2019.06.009 31439436 10.1016/j.clbc.2019.06.009 PMC11372729

[32] Golshan M, Losk K, Mallory MA et al (2016) Implementation of a breast/reconstruction surgery coordinator to reduce preoperative delays for patients undergoing mastectomy with immediate reconstruction. J Oncol Pract 12(3):e338–e343. 10.1200/jop.2015.008672 26883406 10.1200/JOP.2015.008672 PMC4960471

[33] Marks VA, Hsiang WR, Nie J et al (2022) Acceptance of simulated adult patients with Medicaid insurance seeking care in a cancer hospital for a new cancer diagnosis. JAMA Netw Open 5(7):e2222214. 10.1001/jamanetworkopen.2022.22214 35838668 10.1001/jamanetworkopen.2022.22214 PMC9287756

[34] Zipkin RJ, Schaefer A, Wang C et al (2022) Rural-urban differences in breast cancer surgical delays in Medicare beneficiaries. Ann Surg Oncol 29(9):5759–5769. 10.1245/s10434-022-11834-4 35608799 10.1245/s10434-022-11834-4 PMC9128633

[35] Walsh RL, Lofters A, Moineddin R, Krzyzanowska M, Grunfeld E (2021) Primary care continuity and wait times to receiving breast cancer chemotherapy: a population-based retrospective cohort study using CanIMPACT data. Curr Oncol 28(6):4786–4804 34898582 10.3390/curroncol28060405 PMC8628668

[36] Roetzheim RG, Ferrante JM, Lee J-H et al (2012) Influence of primary care on breast cancer outcomes among Medicare beneficiaries. Ann Fam Med 10(5):401–411. 10.1370/afm.1398 22966103 10.1370/afm.1398 PMC3438207

[37] Marzban S, Najafi M, Agolli A, Ashrafi E (2022) Impact of patient engagement on healthcare quality: a scoping review. J Patient Exp 9:23743735221125439. 10.1177/23743735221125439 36134145 10.1177/23743735221125439 PMC9483965

[38] Semprini JT, Devine JW, Lizarraga IM, Charlton ME (2025) Hospital accreditation and geographic disparities in timely cancer care. Health Serv Res. 10.1111/1475-6773.14655 40476571 10.1111/1475-6773.14655 PMC12968059

[39] Khan AA, Shah SK, Naeem W et al (2025) Association between social vulnerability index and time to treatment in resectable non-small cell lung cancer. Ann Thorac Surg. 10.1016/j.athoracsur.2025.04.018 40339972 10.1016/j.athoracsur.2025.04.018

[40] Rasmussen ER, Engelhardt KE (2025) Social vulnerability index: a tool to help close the quality chasm? Ann Thorac Surg. 10.1016/j.athoracsur.2025.05.004 40409353 10.1016/j.athoracsur.2025.05.004

[41] Yan AF, Chen Z, Wang Y et al (2022) Effectiveness of social needs screening and interventions in clinical settings on utilization, cost, and clinical outcomes: a systematic review. Health Equity 6(1):454–475. 10.1089/heq.2022.0010 35801145 10.1089/heq.2022.0010 PMC9257553

[42] Chambers A, Damone E, Chen YT et al (2022) Social support and outcomes in older adults with lung cancer. J Geriatr Oncol 13(2):214–219 34629320 10.1016/j.jgo.2021.09.009 PMC8970686

[43] Milligan M, Erfani P, Orav EJ, Schleicher S, Brooks GA, Lam MB (2024) Practice consolidation among US medical oncologists, 2015–2022. JCO Oncol Pract 20(6):827–834 38408291 10.1200/OP.23.00748 PMC11608122

[44] Raphael MJ, Siemens DR, Booth CM (2019) Would regionalization of systemic cancer therapy improve the quality of cancer care? Proc Am Soc Clin Oncol 15(7):349–356 10.1200/JOP.18.00671 31112481

[45] Kim D, Meyers DJ, Rahman M, Trivedi AN (2021) Comparison of the use of top-ranked cancer hospitals between medicare advantage and traditional medicare. Am J Manag Care 27(10):e355 34668678 10.37765/ajmc.2021.88766 PMC8979370

[46] Raoof M, Jacobson G, Fong Y (2021) Medicare advantage networks and access to high-volume cancer surgery hospitals. Ann Surg 274(4):e315–e319 34506325 10.1097/SLA.0000000000005098

