# Supplementary material for: Delay in breast cancer surgery: evaluating patient, healthcare access, and social vulnerability predictors
Source: Cancer Causes Control. 2026 Feb 10;37(3):49. doi: 10.1007/s10552-026-02127-2 (PMC12891009; doi:10.1007/s10552-026-02127-2)
Supplement: Supplementary file 1 — Supplementary file1 (DOCX 287 KB) [file 10552_2026_2127_MOESM1_ESM.docx]

*Supplementary Information for submission to Cancer Causes & Control*

*Delay in Breast Cancer Surgery: Evaluating Patient, Healthcare Access, and Social Vulnerability Predictors*

*Melanie Boyd, MS^1^; Mandana Rezaeiahari, PhD^1^; Mario Schootman, PhD^2,3,4^;*

*Yong-Moon Park, MD, MS, PhD^1,3^; Kelsey M Owsley, PhD, MPH^1,3^*

*^1^University of Arkansas for Medical Sciences College of Public Health; ^2^University of Arkansas for Medical Sciences College of Medicine; ^3^University of Arkansas for Medical Sciences Winthrop P. Rockefeller Cancer Institute; ^4^University of Arkansas for Medical Sciences College of Nursing*

*Corresponding Author: Melanie Boyd;*

*mboyd@uams.edu;*

*Department of Health Policy and Management*

*Fay W. Boozman College of Public Health, 4301 W. Markham St., #820*

*Little Rock, AR 72205-7199*

**SUPPLEMENTARY INFORMATION**

**Supplemental Table S1. Breast cancer surgery procedure codes**

| **Procedure Codes** |
| --- |
| **CPT: 19110, 19120, 19125, 19126, 19290, 19291, 19160, 19162, 19180, 19182, 19200, 19220, 19240, 19260, 19271, 19272, 19301-19307, S2900** |
| **ICD-9-PCS: 85.20, 85.21, 85.22, 85.23, 85.25, 85.41-85.48, 17.4x** |
| **ICD-10-PCS: 0H5T0ZZ, 0H5T3ZZ, 0H5T7ZZ, 0H5T8ZZ, 0H5TXZZ, 0H5U0ZZ, 0H5U3ZZ, 0H5U7ZZ, 0H5U8ZZ, 0H5UXZZ, 0H5V0ZZ, 0H5V3ZZ, 0H5V7ZZ, 0H5V8ZZ, 0H5VXZZ, 0H5W0ZZ, 0H5W3ZZ, 0H5W7ZZ, 0H5W8ZZ, 0H5WXZZ, 0H5X0ZZ, 0H5X3ZZ, 0H5X7ZZ, 0H5X8ZZ, 0H5XXZZ, 0HBT0Zx, 0HBT3Zx, 0HBT7Zx, 0HBT8Zx, 0HBU0Zx, 0HBU3Zx, 0HBU7Zx, 0HBU8Zx, 0HBUXZx, 0HBV0Zx, 0HBV3Zx, 0HBV7Zx, 0HBV8Zx, 0HBVXZx, 0HBW0Zx, 0HBW3Zx, 0HBW7Zx, 0HBW8Zx, 0HBWXZx, 0HBX0Zx, 0HBX3Zx, 0HBX7Zx, 0HBX8Zx, 0HBXXZx, 0HBY0Zx, 0HBY3Zx, 0HBY7Zx, 0HBY8Zx, 0HBYXZx, 0HPTxxx, 0HPUxxx, 0HTT0ZZ, 0HTU0ZZ, 0HTV0ZZ, 0HTW0ZZ, 0HTX0ZZ, 0HTY0ZZ, 0WB80ZZ, 0WB83ZZ, 0WB84ZZ, 0WB8XZZ, 0KTH0ZZ, 0KTJ0ZZ, 07T50ZZ, 07T60ZZ, 07T70ZZ, 07T80ZZ, 07T90ZZ, 8E0WxCZ** |

**Supplemental Table S2. Characteristics associated with surgical delay >60 days estimated using logistic regression**

|  | |  | |  | **Cancer Stage** | | | | | | | |
| --- | --- | --- | --- | --- | --- | --- | --- | --- | --- | --- | --- | --- |
|  | | **Full Sample** | | **p-value** | **In Situ/**  **Localized** | **p-value** | **Regional/**  **Distant** | | **p-value** | |  |  |
| **Model C-statistic** | | **0.71** | |  | **0.71** |  | **0.73** | |  | |  |  |
| **Patient-level characteristics** | |  | |  |  |  |  | |  | |  |  |
| **Age** | |  | |  |  |  |  | |  | |  |  |
| ***18-49***  ***50-64*** | **Reference**  **0.59**  **(0.47, 0.75)** | | **<0.0001** | | **Reference**  **0.60**  **(0.46, 0.79)** | **0.0004** | | **Reference**  **0.57**  **(0.36, 0.90)** | | **0.02** | |  |
| ***65-74*** | | **0.54**  **(0.39, 0.74)** | | **0.0002** | **0.61**  **(0.44, 0.85)** | **0.0035** | **0.37**  **(0.19, 0.72)** | | **0.004** | |  |  |
| ***75 and older*** | | **0.35**  **(0.24, 0.49)** | | **<0.0001** | **0.37**  **(0.26, 0.54)** | **<0.0001** | **0.28**  **(0.16, 0.48)** | | **<0.0001** | |  |  |
| **Race/ethnicity** | |  | |  |  |  |  | |  | |  |  |
| ***Non-Hispanic White*** | | **Reference** | |  | **Reference** |  | **Reference** | |  | |  |  |
| ***Non-Hispanic Black*** | | **1.82**  **(1.46, 2.29)** | | **<0.0001** | **1.63**  **(1.23, 2.15)** | **0.001** | **2.78***  **(2.01, 3.85)** | | **<0.0001** | |  |  |
| ***Hispanic/Other race*** | | **0.85**  **(0.54, 1.34)** | | **0.48** | **0.87**  **(0.51, 1.47)** | **0.60** | **0.87**  **(0.28, 2.72)** | | **0.82** | |  |  |
| **Insurance** | |  | |  |  |  |  | |  | |  |  |
| ***Private*** | | **Reference** | |  | **Reference** |  | **Reference** | |  | |  |  |
| ***Medicaid*** | | **1.27**  **(0.92, 1.76)** | | **0.15** | **1.44**  **(1.00, 2.06)** | **0.049** | **0.97**  **(0.51, 1.86)** | | **0.93** | |  |  |
| ***Medicare*** | | **0.89**  **(0.69, 1.14)** | | **0.34** | **0.84**  **(0.66, 1.06)** | **0.14** | **0.99**  **(0.57, 1.72)** | | **0.97** | |  |  |
| ***Medicare/Medicaid*** | | **1.01**  **(0.66, 1.52)** | | **0.98** | **1.09**  **(0.67, 1.78)** | **0.73** | **0.84**  **(0.40, 1.75)** | | **0.64** | |  |  |
| **Cancer Stage**  ***In situ/Localized*** | | **Reference** | |  |  |  |  | |  | |  |  |
| ***Regional/Distant*** | | **1.18**  **(0.95, 1.47)** | | **0.13** | **--** |  | **--** | | **--** | |  |  |
| **Rural** | | **0.89**  **(0.65, 1.21)** | | **0.43** | **0.97**  **(0.66, 1.42)** | **0.88** | **0.68**  **(0.39, 1.19)** | | **0.17** | |  |  |
| **Triple-negative** | | **0.97**  **(0.73, 1.28)** | | **0.81** | **0.89**  **(0.60, 1.31)** | **0.54** | **1.21**  **(0.76, 1.92)** | | **0.42** | |  |  |
| **Mastectomy** | | **3.31**  **(2.77, 3.95)** | | **<0.0001** | **3.44**  **(2.70, 4.38)** | **<0.0001** | **2.97**  **(2.01, 4.39)** | | **<0.0001** | |  |  |
| **County-level characteristics** | |  | |  |  |  |  | |  | |  |  |
| **% adults with routine**  **doctor visit** | | **0.89**  **(0.82, 0.97)** | | **0.01** | **0.94**  **(0.85, 1.04)** | **0.20** | **0.77***  **(0.68, 0.86)** | | **<0.001** | |  |  |
| **Hospital w/ oncology care** | | **0.87**  **(0.64, 1.17)** | | **0.35** | **0.78**  **(0.54, 1.13)** | **0.19** | **1.15**  **(0.72, 1.83)** | | **0.55** | |  |  |
| **Hospital w/ surgical services** | | **1.31**  **(1.00, 1.71)** | | **0.05** | **1.16**  **(0.86, 1.57)** | **0.34** | **1.95**  **(1.05, 3.64)** | | **0.04** | |  |  |
| **Commission on Cancer Facility** | | **1.13**  **(0.83, 1.53)** | | **0.43** | **0.94**  **(0.69, 1.28)** | **0.70** | **1.92***  **(1.16, 3.20)** | | **0.01** | |  |  |
| **Socioeconomic**  **SVI - *Top quartile*** | | **1.04**  **(0.74, 1.45)** | | **0.82** | **0.86**  **(0.59, 1.25)** | **0.42** | **1.79***  **(0.98, 3.28)** | | **0.06** | |  |  |
| **Household**  **SVI - *Top quartile*** | | **1.02**  **(0.78, 1.33)** | | **0.89** | **0.95**  **(0.70, 1.29)** | **0.74** | **1.23**  **(0.81, 1.85)** | | **0.33** | |  |  |
| **Minority status**  **SVI - *Top quartile*** | | **1.26**  **(0.92, 1.71)** | | **0.15** | **1.32**  **(0.91, 1.91)** | **0.14** | **1.04**  **(0.68, 1.59)** | | **0.86** | |  |  |
| **Housing/transportation**  **SVI - *Top quartile*** | | **1.38**  **(1.06, 1.81)** | | **0.02** | **1.56**  **(1.12, 2.18)** | **0.01** | **1.03**  **(0.75, 1.41)** | | **0.86** | |  |  |
| **Year** | |  | |  |  |  |  | |  | |  |  |
| ***2013*** | | **Reference** | |  | **Reference** |  | **Reference** | |  | |  |  |
| ***2014*** | | **1.04**  **(0.75, 1.45)** | | **0.81** | **1.02**  **(0.70, 1.47)** | **0.94** | **1.10**  **(0.51, 2.38)** | | **0.80** | |  |  |
| ***2015*** | | **1.17**  **(0.83, 1.64)** | | **0.38** | **1.10**  **(0.77, 1.56)** | **0.60** | **1.34**  **(0.71, 2.51)** | | **0.36** | |  |  |
| ***2016*** | | **1.19**  **(0.84, 1.70)** | | **0.33** | **1.17**  **(0.77, 1.79)** | **0.45** | **1.25**  **(0.59, 2.62)** | | **0.55** | |  |  |
| ***2017*** | | **1.48**  **(1.07, 2.05)** | | **0.02** | **1.26**  **(0.88, 1.79)** | **0.20** | **2.40**  **(1.21, 4.74)** | | **0.01** | |  |  |
| ***2018*** | | **1.49**  **(1.05, 2.12)** | | **0.03** | **1.38**  **(0.97, 1.95)** | **0.07** | **1.99**  **(0.85, 4.65)** | | **0.11** | |  |  |
| ***2019*** | | **2.20**  **(1.55, 3.11)** | | **<.0001** | **2.01**  **(1.34, 3.01)** | **0.001** | **3.11**  **(1.58, 6.11)** | | **0.001** | |  |  |

Notes: Analyses used logistic regression with standard errors clustered at the county-levels. *Indicates significant difference (p<0.05) by cancer stage using fully interacted models.

**Supplemental Table S3. Characteristics associated with time-to-surgery >45 days estimated using logistic regression**

|  | |  | |  | **Cancer Stage** | | | | | | | |
| --- | --- | --- | --- | --- | --- | --- | --- | --- | --- | --- | --- | --- |
|  | | **Full Sample** | | **p-value** | **In Situ/**  **Localized** | **p-value** | **Regional/**  **Distant** | | **p-value** | |  |  |
| **Model C-statistic** | | **0.68** | |  | **0.68** |  | **0.69** | |  | |  |  |
| **Patient-level characteristics** | |  | |  |  |  |  | |  | |  |  |
| **Age** | |  | |  |  |  |  | |  | |  |  |
| ***18-49***  ***50-64*** | **Reference**  **0.69**  **(0.59, 0.80)** | | **<0.0001** | | **Reference**  **0.68**  **(0.56, 0.82)** | **0.0001** | | **Reference**  **0.73**  **(0.52, 1.03)** | | **0.07** | |  |
| ***65-74*** | | **0.64**  **(0.51, 0.80)** | | **0.0002** | **0.66**  **(0.52, 0.83)** | **0.001** | **0.56**  **(0.34, 0.95)** | | **0.03** | |  |  |
| ***75 and older*** | | **0.46**  **(0.36, 0.59)** | | **<0.0001** | **0.49**  **(0.37, 0.66)** | **<0.0001** | **0.38**  **(0.24, 0.61)** | | **0.001** | |  |  |
| **Race/ethnicity** | |  | |  |  |  |  | |  | |  |  |
| ***Non-Hispanic White*** | | **Reference** | |  | **Reference** |  | **Reference** | |  | |  |  |
| ***Non-Hispanic Black*** | | **1.45**  **(1.23, 1.70)** | | **<0.0001** | **1.27**  **(1.03, 1.57)** | **0.03** | **2.34***  **(1.65, 3.33)** | | **<0.001** | |  |  |
| ***Hispanic/Other race*** | | **0.93**  **(0.59, 1.47)** | | **0.74** | **0.81**  **(0.49, 1.36)** | **0.43** | **1.49**  **(0.66, 3.38)** | | **0.34** | |  |  |
| **Insurance** | |  | |  |  |  |  | |  | |  |  |
| ***Private*** | | **Reference** | |  | **Reference** |  | **Reference** | |  | |  |  |
| ***Medicaid*** | | **1.43**  **(1.11, 1.86)** | | **0.006** | **1.68**  **(1.20, 2.36)** | **0.003** | **0.95**  **(0.50, 1.79)** | | **0.86** | |  |  |
| ***Medicare*** | | **0.93**  **(0.77, 1.11)** | | **0.40** | **0.87**  **(0.74, 1.02)** | **0.08** | **1.12**  **(0.71, 1.78)** | | **0.62** | |  |  |
| ***Medicare/Medicaid*** | | **0.99**  **(0.76, 1.30)** | | **0.96** | **1.02**  **(0.71, 1.46)** | **0.92** | **0.99**  **(0.55, 1.80)** | | **0.98** | |  |  |
| **Cancer Stage** | |  | |  |  |  |  | |  | |  |  |
| ***In Situ/ Localized*** | | **Reference** | |  | **--** |  | **--** | | **--** | |  |  |
| ***Regional/Distant*** | | **0.96**  **(0.80, 1.15)** | | **0.65** | **--** |  | **--** | | **--** | |  |  |
| **Rural** | | **0.99**  **(0.77, 1.27)** | | **0.93** | **1.09**  **(0.82, 1.45)** | **0.53** | **0.72**  **(0.47, 1.10)** | | **0.13** | |  |  |
| **Triple-negative** | | **1.00**  **(0.77, 1.29)** | | **0.98** | **0.97**  **(0.73, 1.28)** | **0.81** | **1.10**  **(0.69, 1.77)** | | **0.68** | |  |  |
| **Mastectomy** | | **3.07**  **(2.65, 3.57)** | | **<0.0001** | **3.18**  **(2.68, 3.79** | **<0.0001** | **2.77**  **(2.14, 3.59)** | | **<0.001** | |  |  |
| **County-level characteristics** | |  | |  |  |  |  | |  | |  |  |
| **% adults with routine**  **doctor visit** | | **0.93**  **(0.85, 1.02)** | | **0.11** | **0.97**  **(0.89, 1.06)** | **0.51** | **0.80***  **(0.69, 0.93)** | | **0.004** | |  |  |
| **Hospital w/ oncology care** | | **0.85**  **(0.67, 1.09)** | | **0.20** | **0.78**  **(0.59, 1.03)** | **0.08** | **1.12**  **(0.77, 1.64)** | | **0.56** | |  |  |
| **Hospital w/ surgical services** | | **1.18**  **(0.92, 1.52)** | | **0.20** | **1.08**  **(0.82, 1.41)** | **0.58** | **1.63**  **(0.97, 2.73)** | | **0.06** | |  |  |
| **Commission on Cancer hospital** | | **0.94**  **(0.65, 1.36)** | | **0.75** | **0.86**  **(0.62, 1.19)** | **0.35** | **1.39**  **(0.62, 3.13)** | | **0.42** | |  |  |
| **Socioeconomic**  **SVI - *Top quartile*** | | **1.00**  **(0.75, 1.33)** | | **0.97** | **0.86**  **(0.63, 1.16)** | **0.31** | **1.55***  **(0.93, 2.59)** | | **0.09** | |  |  |
| **Household**  **SVI - *Top quartile*** | | **0.90**  **(0.72, 1.14)** | | **0.39** | **0.88**  **(0.68, 1.13)** | **0.31** | **0.99**  **(0.68, 1.46)** | | **0.97** | |  |  |
| **Minority status**  **SVI - *Top quartile*** | | **1.16**  **(0.82, 1.66)** | | **0.40** | **1.16**  **(0.81, 1.65)** | **0.41** | **1.19**  **(0.70, 2.02)** | | **0.53** | |  |  |
| **Housing/transportation**  **SVI - *Top quartile*** | | **1.27**  **(0.96, 1.67)** | | **0.09** | **1.34**  **(0.99, 1.82)** | **0.06** | **1.11**  **(0.75, 1.66)** | | **0.59** | |  |  |
| **Year** | |  | |  |  |  |  | |  | |  |  |
| **2013** | | **Reference** | |  | **Reference** |  | **Reference** | |  | |  |  |
| **2014** | | **1.01**  **(0.78, 1.32)** | | **0.92** | **1.06**  **(0.81, 1.38)** | **0.67** | **0.80**  **(0.44, 1.46)** | | **0.46** | |  |  |
| **2015** | | **1.24**  **(0.96, 1.61)** | | **0.10** | **1.24**  **(0.93, 1.66)** | **0.13** | **1.11**  **(0.69, 1.80)** | | **0.66** | |  |  |
| **2016** | | **1.29**  **(0.97, 1.71)** | | **0.08** | **1.23**  **(0.91, 1.65)** | **0.18** | **1.35**  **(0.80, 2.28)** | | **0.25** | |  |  |
| **2017** | | **1.62**  **(1.21, 2.17)** | | **0.002** | **1.58**  **(1.13, 2.21)** | **0.01** | **1.67**  **(1.03, 2.72)** | | **0.04** | |  |  |
| **2018** | | **1.59**  **(1.21, 2.09)** | | **0.001** | **1.68**  **(1.25, 2.26)** | **0.001** | **1.21**  **(0.67, 2.18)** | | **0.52** | |  |  |
| **2019** | | **2.13**  **(1.58, 2.87)** | | **<0.0001** | **2.15**  **(1.55, 2.98)** | **<0.001** | **1.97**  **(1.15, 3.38)** | | **0.01** | |  |  |

Notes: Analyses used logistic regression with standard errors clustered at the county-levels. *Indicates significant difference (p<0.05) by cancer stage using fully interacted models.

**Supplemental Figure S1: Number of breast cancer patients in study sample by county**


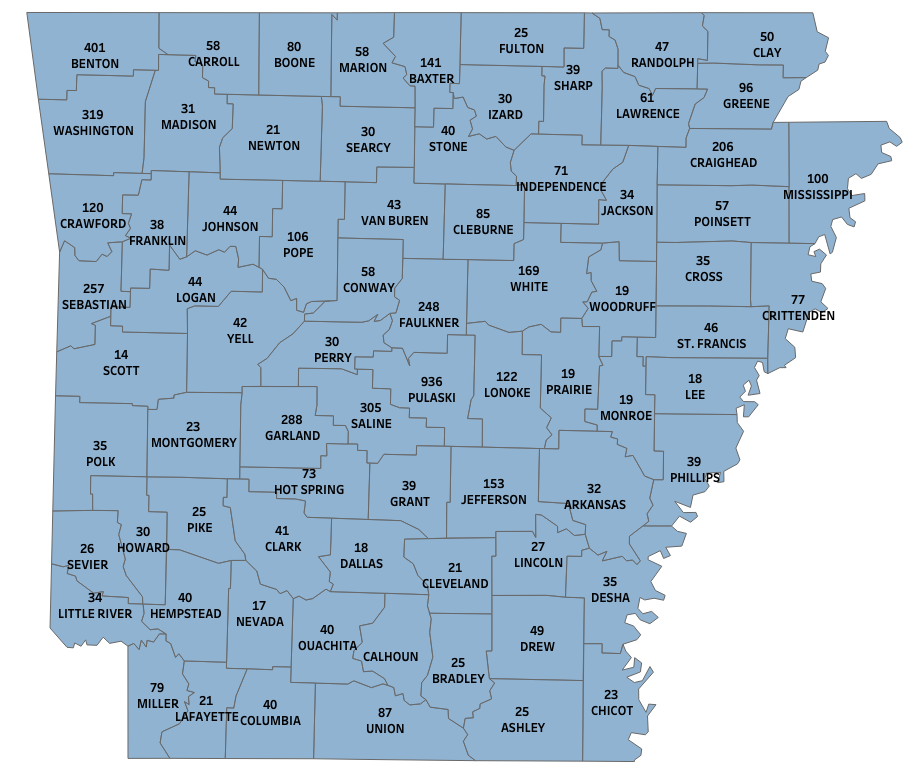


Note: Counties with less than 10 cases are excluded.

**Supplemental Table S4. Characteristic groupings associated with surgical delay >60 days estimated using logistic regression**

|  | **Full Sample** | **Patient Only** | **Healthcare Access Only** | **SVI Only** | **Patient/**  **Access** | **Patient/**  **SVI** | **Access/**  **SVI** |
| --- | --- | --- | --- | --- | --- | --- | --- |
| **Model C-statistic** | **0.71** | **0.70** | **0.56** | **0.56** | **0.71** | **0.71** | **0.57** |
| **Predictor Variables** |  |  |  |  |  |  |  |
| **Age**  ***18-49***  ***50-64*** | **Reference**  **0.59**  **(0.47, 0.75)** | **Reference**  **0.60**  **(0.48, 0.74)** |  |  | **Reference**  **0.59**  **(0.47, 0.75)** | **Reference**  **0.60**  **(0.48, 0.75)** |  |
| ***65-74*** | **0.54**  **(0.39, 0.74)** | **0.55**  **(0.40, 0.75)** |  |  | **0.54**  **(0.40, 0.75)** | **0.55**  **(0.40, 0.75)** |  |
| ***75 and older*** | **0.35**  **(0.24, 0.49)** | **0.34**  **(0.24, 0.48)** |  |  | **0.34**  **(0.24, 0.49)** | **0.35**  **(0.25, 0.50)** |  |
| **Race/ethnicity** |  |  |  |  |  |  |  |
| ***Non-Hispanic White*** | **Reference** | **Reference** |  |  | **Reference** | **Reference** |  |
| ***Non-Hispanic Black*** | **1.82**  **(1.46, 2.29)** | **1.56**  **(1.22, 2.00)** |  |  | **1.91**  **(1.51, 2.41)** | **1.54**  **(1.19, 1.98)** |  |
| ***Hispanic/Other race*** | **0.85**  **(0.54, 1.34)** | **0.81**  **(0.51, 1.30)** |  |  | **0.86**  **(0.55, 1.36)** | **0.79**  **(0.50, 1.26)** |  |
| **Insurance** |  |  |  |  |  |  |  |
| ***Private*** | **Reference** | **Reference** |  |  | **Reference** | **Reference** |  |
| ***Medicaid*** | **1.27**  **(0.92, 1.76)** | **1.30**  **(0.94, 1.81)** |  |  | **1.28**  **(0.92, 1.78)** | **1.31**  **(0.95, 1.81)** |  |
| ***Medicare*** | **0.89**  **(0.69, 1.14)** | **0.89**  **(0.69, 1.14)** |  |  | **0.89**  **(0.69, 1.14)** | **0.88**  **(0.69, 1.14)** |  |
| ***Medicare/Medicaid*** | **1.01**  **(0.66, 1.52)** | **1.01**  **(0.67, 1.51)** |  |  | **1.01**  **(0.67, 1.52)** | **1.02**  **(0.67, 1.54)** |  |
| **Cancer Stage**  ***In situ/Localized*** | **Reference** | **Reference** |  |  | **Reference** | **Reference** |  |
| ***Regional/Distant*** | **1.18**  **(0.95, 1.47)** | **1.16**  **(0.92, 1.46)** |  |  | **1.19**  **(0.95, 1.48)** | **1.15**  **(0.92, 1.44)** |  |
| **Rural** | **0.89**  **(0.65, 1.21)** | **0.87**  **(0.64, 1.18)** |  |  | **0.85**  **(0.63, 1.14)** | **0.99**  **(0.73, 1.36)** |  |
| **HER2-negative** | **0.97**  **(0.73, 1.28)** | **0.95**  **(0.72, 1.26)** |  |  |  | **0.96**  **(0.73, 1.26)** |  |
| **Mastectomy** | **3.31**  **(2.77, 3.95)** | **3.35**  **(2.80, 4.01)** |  |  |  | **3.37**  **(2.80, 4.06)** |  |
| **% adults with routine**  **doctor visit** | **0.89**  **(0.82, 0.97)** |  | **0.97**  **(0.85, 1.10)** |  | **0.90**  **(0.80, 1.01)** |  | **0.95**  **(0.87, 1.04)** |
| **Hospital w/ oncology care** | **0.87**  **(0.64, 1.17)** |  | **0.96**  **(0.64, 1.45)** |  | **1.01**  **(0.71, 1.44)** |  | **0.78**  **(0.57, 1.06)** |
| **Hospital w/ surgical services** | **1.31**  **(1.00 1.71)** |  | **1.26**  **(0.94, 1.67)** |  | **1.26**  **(0.94, 1.69)** |  | **1.31**  **(1.00, 1.70)** |
| **Commission on Cancer Facility** | **1.13**  **(0.83, 1.53)** |  | **1.17**  **(0.76, 1.79)** |  | **1.14**  **(0.78, 1.67)** |  | **1.11**  **(0.77, 1.60)** |
| **Socioeconomic**  **SVI - *Top quartile*** | **1.04**  **(0.74, 1.45)** |  |  | **0.98**  **(0.74, 1.31)** |  | **0.89**  **(0.63, 1.26)** | **1.00**  **(0.77, 1.31)** |
| **Household**  **SVI - *Top quartile*** | **1.02**  **(0.78, 1.33)** |  |  | **1.07**  **(0.81, 1.42)** |  | **0.99**  **(0.76, 1.28)** | **1.07**  **(0.82, 1.40)** |
| **Minority status**  **SVI - *Top quartile*** | **1.26**  **(0.92, 1.71)** |  |  | **1.22**  **(0.83, 1.78)** |  | **1.15**  **(0.76, 1.73)** | **1.38**  **(0.95, 1.98)** |
| **Housing/transportation**  **SVI - *Top quartile*** | **1.38**  **(1.06, 1.81)** |  |  | **1.39**  **(0.95, 2.03)** |  | **1.37**  **(0.93, 2.02)** | **1.50**  **(1.09, 2.06)** |
| **Year** |  |  |  |  |  |  |  |
| ***2013*** | **Reference** | **Reference** | **Reference** | **Reference** | **Reference** | **Reference** | **Reference** |
| ***2014*** | **1.04**  **(0.75, 1.45)** | **1.06**  **(0.77, 1.45)** | **1.05**  **(0.77, 1.44)** | **1.05**  **(0.77, 1.45)** | **1.04**  **(0.75, 1.44)** | **1.06**  **(0.76, 1.47)** | **1.05**  **(0.77, 1.44)** |
| ***2015*** | **1.17**  **(0.83, 1.64)** | **1.23**  **(0.90, 1.68)** | **1.18**  **(0.87, 1.59)** | **1.19**  **(0.87, 1.61)** | **1.18**  **(0.85, 1.64)** | **1.21**  **(0.88, 1.68)** | **1.16**  **(0.85, 1.59)** |
| ***2016*** | **1.19**  **(0.84, 1.70)** | **1.27**  **(0.89, 1.81)** | **1.16**  **(0.83, 1.62)** | **1.15**  **(0.82, 1.62)** | **1.21**  **(0.85, 1.72)** | **1.26**  **(0.88, 1.79)** | **1.12**  **(0.80, 1.58)** |
| ***2017*** | **1.48**  **(1.07, 2.05)** | **1.60**  **(1.15, 2.23)** | **1.38**  **(1.01, 1.88)** | **1.36**  **(1.00, 1.84)** | **1.53**  **(1.11, 2.12)** | **1.57**  **(1.12, 2.18)** | **1.31**  **(0.97, 1.77)** |
| ***2018*** | **1.49**  **(1.05, 2.12)** | **1.60**  **(1.13, 2,28)** | **1.40**  **(1.01, 1.93)** | **1.39**  **(1.02, 1.90)** | **1.53**  **(1.07, 2.20)** | **1.57**  **(1.11, 2.22)** | **1.34**  **(0.98, 1.83)** |
| ***2019*** | **2.20**  **(1.55, 3.11)** | **2.15**  **(1.56, 2.97)** | **1.76**  **(1.21, 2.56)** | **1.69**  **(1.21, 2.34)** | **2.26**  **(1.57, 3.25)** | **2.12**  **(1.52, 2.94)** | **1.70**  **(1.20, 2.41)** |

Notes: Analyses used logistic regression with standard errors clustered at the county-levels.
